# Fabrication of ZnO Thin Films Doped with Na at Different Percentages for Sensing CO_2_ in Small Quantities at Room Temperature

**DOI:** 10.3390/s25092705

**Published:** 2025-04-24

**Authors:** Marina Stramarkou, Achilleas Bardakas, Magdalini Krokida, Christos Tsamis

**Affiliations:** 1 School of Chemical Engineering, National Technical University of Athens (NTUA), 15772 Athens, Greece; mkrok@chemeng.ntua.gr; 2 Institute of Nanoscience and Nanotechnology, National Centre for Scientific Research (NCSR) “Demokritos”, 15341 Athens, Greece; a.bardakas@inn.demokritos.gr (A.B.); c.tsamis@inn.demokritos.gr (C.T.)

**Keywords:** chemical sensors, food packaging, food safety, nanotechnology

## Abstract

The objective of this study is the fabrication of sensors which can detect modifications in CO_2_ concentrations at room temperature, thus indicating the quality or microbial spoilage of food products when incorporated into food packaging. ZnO nanostructures are known for their ability to detect organic gases; however, their effectiveness is limited to high temperatures (greater than 200 °C). To overcome this limitation, sodium (Na) doping is investigated as a way to enhance the sensing properties of ZnO films and lower the working temperature. In this study, undoped and Na-doped ZnO thin films were developed via the sol-gel method with different Na percentages (2.5, 5 and 7.5%) and were deposited via spin coating. The crystal structure, the morphology, and the surface topography of the developed films were characterized by X-ray Diffraction (XRD), Scanning Electron Microscopy (SEM), and Atomic Force Microscopy (AFM), respectively. Furthermore, the response to CO_2_ was measured by varying its concentration up to 500 ppm at room temperature. All the developed films presented the characteristic diffraction peaks of the ZnO wurtzite hexagonal crystal structure. SEM revealed that the films consisted of densely packed grains, with an average particle size of 58 nm. Na doping increased the film thickness but reduced the surface roughness. Finally, the developed sensors demonstrated very good CO_2_ sensing properties, with the 2.5% Na-doped sensor having an enhanced sensing performance concerning sensitivity, response, and recovery times. This leads to the conclusion that Na-doped ZnO sensors could be used for the detection of microbial spoilage in food products at room temperature, making them suitable for smart food packaging applications.

## 1. Introduction

Carbon dioxide (CO_2_) is a microbial specific marker, as it is a product of the metabolic activities of microbes contaminating food products after unsuitable manipulation and storage and causing food deterioration and, potentially, disease [1]. Specifically, the activity of many microbes that degrade and spoil fish, meat, grains [2], fruits, vegetables, and their juices [3] leads to the formation of CO_2_, which can change their characteristics and have harmful effects [4]. In addition, CO_2_ is also a preservative at high concentrations in modified atmosphere food packaging. Therefore, it can indicate not only food spoilage but also the integrity of the packaging. Thus, sensors that detect its presence or changes in its concentration can indicate the quality, freshness, and shelf life of food products [5,6].

In recent years, a variety of CO_2_ sensors have been developed based on different detection principles, such as optical absorption, electrical resistance, amperometry, etc. However, the practical use of conventional CO_2_ sensors in food is still limited, as they exhibit several disadvantages, such as high cost, large weight and size, and low durability [7,8,9]. To enable the widespread adoption of CO_2_ sensors, economical devices that offer simplicity, accuracy, and reliability are needed. To meet these needs, there is an increasing interest in nanomaterial-based sensors due to their cost-effectiveness, robustness to harsh conditions, and stability. Nanomaterials are commonly used for the fabrication of CO_2_ sensors thanks to their high surface area, which facilitates the adsorption of gas molecules and improves their sensitivity [10,11].

Based on the detection mechanism, CO_2_ nano-sensors can be categorized into electrochemical, such as chemi-resisitive, capacitive and inductive sensors, and optical, such as non-dispersive infrared, colorimetric, and refractometric sensors [8]. Sensors using electrical transducers are convenient for food applications, as they are fast, economical, and promise easy integration into food packaging materials [6,9].

Electrochemical sensors able to monitor the modification, and more specifically, the increase in the concentration of CO_2_ can specify the freshness and the quality of food products [5]. With a view to extending the shelf life of fresh and packaged food products, it is beneficial to detect the concentration of CO_2_ using chemical sensors [12].

Out of the large pool of nanomaterials that could be used for the development of CO_2_ chemical sensors, zinc oxide (ZnO) is commonly examined [13,14]. ZnO is a II–VI group semiconductor with unique characteristics, such as fast response, low detection limit, high selectivity, and reliable performance. Those properties enable its efficient utilization in various applications, such as chemical sensors for the detection of gases [15].

Although many reports have indicated that ZnO nanostructures can be used for the detection of organic gases, most of them only worked at temperatures greater than 200 °C [11,16]. CO_2_ is an inert gas with limited oxidizing and reducing properties, which results in poor sensing performance when using semiconductor sensors [17]. This fact critically limits their application in food packaging, where detection temperatures that are as low as possible are required, with ambient temperature being ideal.

Doping ZnO with suitable materials, such as metal atoms and metal oxides like gold (Au) [18], cadmium (Cd) [12], lanthanum (La) [19], aluminum (Al) [20], calcium (Ca) [21], etc. is a method to enhance the sensing behavior of ZnO thin films, lowering the working temperature and at the same time achieving high reliability [20,22,23]. Via the suitable doping of ZnO with metals, its properties can be changed and regulated [24]. The replacement of Zn^2+^ ions can occur through either lower (group 1) or higher (group 3) valence ion dopants, which increase carrier concentration and lower resistivity (p- or n-type doping). Alternatively, isovalent dopants (i.e., Ca^2+^) with larger ionic radii can be added to distort the lattice and improve CO_2_ adsorption [9,25].

Sodium (Na) is a suitable group 1 acceptor with a relatively high hole concentration and shallow substitutional level. Due to its shallow acceptor production, Na is an excellent substitute for Zn as a p-type dopant for ZnO and has gained interest, making it a promising candidate for the fabrication of various p-n junction devices [23,26]. Na also reduces oxygen vacancy density, which is critical in achieving stable p-type conduction [27].

To date, various types of CO_2_ chemi-resistive sensors based on ZnO thin film structures have been fabricated employing a range of fabrication methods, adjusting different process parameters, and doping with one or more materials in order to enhance the CO_2_ sensing features. However, as shown in Table 1, most of these developed sensors are limited in one of two ways: they can either detect CO_2_ only at high concentrations (>1000 ppm) [21,28,29,30] or they require elevated temperatures (>100 °C) to detect lower concentrations [12,31,32,33].

The objective of this study is the fabrication of undoped ZnO and 2.5, 5, and 7.5% Na-doped ZnO thin films, which can sense CO_2_ gas in low concentrations at room temperature (25 °C), thus being able to be incorporated into food packaging and to inform about the quality of food products. The crystal structure of the developed films was characterized by X-ray Diffraction (XRD), their morphology and thickness were investigated through Field Emission Scanning Electron Microscopy (FE-SEM), and their surface topography was evaluated using Atomic Force Microscopy (AFM). The response to CO_2_ was measured by modulating its concentration up to 500 ppm at room temperature using an appropriate experimental setup. To the best of our knowledge, this is the first work published that studied ZnO sensors which are able to detect CO_2_ at very low concentrations, at room temperature, and without the use of UV light, for food packaging applications.

## 2. Materials and Methods

### 2.1. Sol-Gel Preparation, Film Deposition, and Substrate Development

The preparation of the sol-gels, as well as the deposition of the films, were based on the study of Basyooni et al. (2017) and further expanded regarding the concentration envelope of the dopant, including the percentages of 5 and 7.5% Na, using a SiO_2_ substrate [23].

The preparation of the undoped ZnO sol-gels involved the dissolution of zinc acetate dihydrate (source material) (Sigma-Aldrich, Darmstadt, Germany) in 2-methoxyethanol (2ME) solvent (Sigma-Aldrich, Darmstadt, Germany) in order to obtain a 0.5 M solution, with the addition of 0.5 M ethanolamine (MEA) (Sigma-Aldrich, Darmstadt, Germany) as a stabilizing agent. For the preparation of the Na-doped sol-gels, sodium acetate (dopant material) (Sigma-Aldrich, Darmstadt, Germany) was added to the above solutions, reaching a final concentration of 2.5, 5 and 7.5 at % Na. The solutions were magnetically stirred for 120 min at 60 °C and were left at room-temperature overnight in a dark environment.

### 2.2. Sensor Fabrication

**Figure 1:**
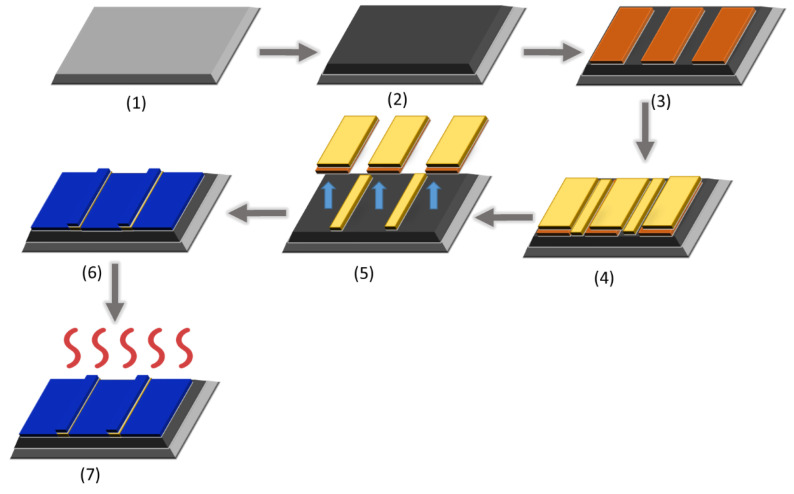
CO_2_ sensor fabrication process steps: (**1**) cleaning of Si wafer with piranha solution, (**2**) thermal oxidation, (**3**) negative lithography, (**4**) deposition of Ti/Au thin film, (**5**) lift-off process, (**6**) ZnO thin film deposition, and (**7**) final annealing (explanation of colors: light grey: Si wafer; dark grey: oxidized Si wafer; orange: Au, yellow: Ti/Au thin film, blue: ZnO thin film).

The process started with the cleaning of a four-inch n-type Si (100) wafer using a piranha solution (H_2_O_2_/H_2_SO_4_ (VSLI-grade, Microchemicals GmbH, Ulm, Germany), 1:1 vol.) (Figure 1, step 1) in order to remove possible organic contamination, followed by rinsing with deionized (DI) water and blow drying with nitrogen (N_2_). After cleaning, a 100 nm-thick silicon dioxide (SiO_2_) was grown on the wafer using dry thermal oxidation, followed by another cleaning step before further processing (Figure 1, step 2). Subsequently, negative lithography was carried out by spin coating a 1.3 μm thick AZ-5214E (Microchemicals GmbH, Ulm, Germany) photo-resistant thin film on the SiO_2_ at 5000 rpm, followed by a pre-bake at 110 °C for 90 s. Exposure under UV light was performed using a SUSS Microtec MA6 mask aligner for 500 s (Step 3), using a quartz photomask containing the device features. After exposure, a post-exposure bake was performed at 120 °C for 90 s. Following that, a flood exposure was performed for 3 min in order to perform the image reversal process. The development of the exposed pattern was performed by soaking the wafer in the AZ-726 MIF (VLSI-grade, Microchemicals GmbH, Ulm, Germany) developer for 60 s. Afterward, direct current (DC) magnetron sputtering was employed in order to deposit a 10 nm thick titanium (Ti) adhesion layer and a 50 nm thick gold (Au) layer (Figure 1, step 4). Following the deposition, the lift-off process (Figure 1, step 5) was performed in an acetone (EMSURE^®^ ACS, ISO, Reag. Ph Eur, Merck KGaA, Darmstadt, Germany) bath for 15 min without ultrasonication, defining the final interdigitated electrode (IDE) geometry, followed by rinsing with isopropanol (EMSURE^®^ ACS, ISO, Reag. Ph Eur, Merck KGaA, Darmstadt, Germany) and DI water and blow drying with N_2_. Each sample was cleaved from the processed wafer using a diamond scribe containing three sensors. The final fabrication step was the deposition of the ZnO thin films using spin coating. Prior to the deposition, the area around the IDEs was masked using Kapton^®^ tape in order to deposit the ZnO thin film only at the electrode area. The film deposition was carried out with seven spin coatings (2000 rpm, 30 s) (Figure 1, step 6), followed by thermal treatments (after each coating) using a hotplate (180 °C, 20 min) and a final annealing step at 500 °C for 120 min (Figure 1, step 7). The steps of the whole fabrication process are shown schematically in Figure 1.

### 2.3. Morphological and Crystallograpic Characterisation

X-ray diffraction (XRD) analysis was used to investigate the microstructure of the doped and undoped ZnO thin film (D8 Advance Bruker, Billerica, MA, USA) using a Cu-Kα (λ = 1.5405 nm) radiation source at a 0.029/s scanning rate in the 2θ range of 20–80 degrees. The crystallite size of the films was calculated using Debye Scherrer’s formula:

(1) $$D=\frac{0.94\lambda }{\beta \;\cos\theta }$$

where *λ* is the wavelength of the X-ray source, *β* is the full width at half maximum (FWHM) in radians, and *θ* is the diffraction angle of the highest peak in radians.

The morphological properties and thickness of the films were studied using field emission-scanning electron microscopy (FE-SEM, JEOL JSM-7401F, Tokyo, Japan) operating at a voltage of 2.5 kV in top-view and cross-sectional perspective. The analysis of FE-SEM micrographs was performed with the open-source image analysis tool ImageJ (v. 1.54g).

Surface roughness measurements were performed by atomic force microscopy (AFM) (NTEGRA Prima atomic force microscope, (Spectrum Instruments Ltd., Limerick, Ireland)) in intermittent contact mode. For this purpose, NT-MDT NSGO1 silicon, N-type, antimony doped cantilevers with Au reflective coating and a nominal force constant of 5.1 N/m were used. AFM images were created and analyzed with the software Gwyddion 2.60 (Free and Open-Source software, Department of Nanometrology, Czech Metrology Institute).

A statistical analysis was performed with one-way and factorial analysis of variance (ANOVA) in order to analyze the differences between the four developed sensors concerning their particle size and thickness using STATISTICA software (v.13.6 StatSoft^®^Inc., Palo Alto, CA, USA).

### 2.4. Electrical Characterization

The sensing performance of the developed sensors was evaluated at different CO_2_ concentrations: 50, 125, 250, 330, and 500 ppm. After the fabrication process, the sample containing the three CO_2_ sensors was mounted on a printed circuit board (PCB) and connected via wire bonding. During the measurements, the sensors were connected to a Keithley 2400 (Tektronix, Berkshire, UK) source measure unit (SMU) and placed in a sealed Teflon^®^ (Manchester, UK) chamber at room temperature (25 °C). The control of the CO_2_ content was done by providing a mixture of synthesized dry air (N_2_ 80%, O_2_ 20%) and N_2_ gas with 0.1% CO_2_ through Brooks^®^ 5800-S mass flow controllers (MFC) (Brooks Instrument, Hatfield, PA, USA). A bias of 2 V was constantly supplied by the SMU, and the current of the sensor was recorded. The response (%) of the sensors was calculated as:

(2) $$S\left(\%\right)=\frac{R_{a}-R_{g}}{R_{a}}\times \;100$$

where *S* is response, *R_a_* is the resistance in the absence of CO_2_, and *R_g_* is the resistance in the presence of different concentrations of CO_2_. The response time was equal to the time the sensors needed to achieve 90% of the total signal change from the moment they were exposed to a specific CO_2_ concentration. The recovery time was equal to the time required for the sensors’ signal to return to 90% of their initial value after the CO_2_ concentration value was zero [47].

## 3. Results

### 3.1. X-Ray Diffraction (XRD)

**Figure 2:**
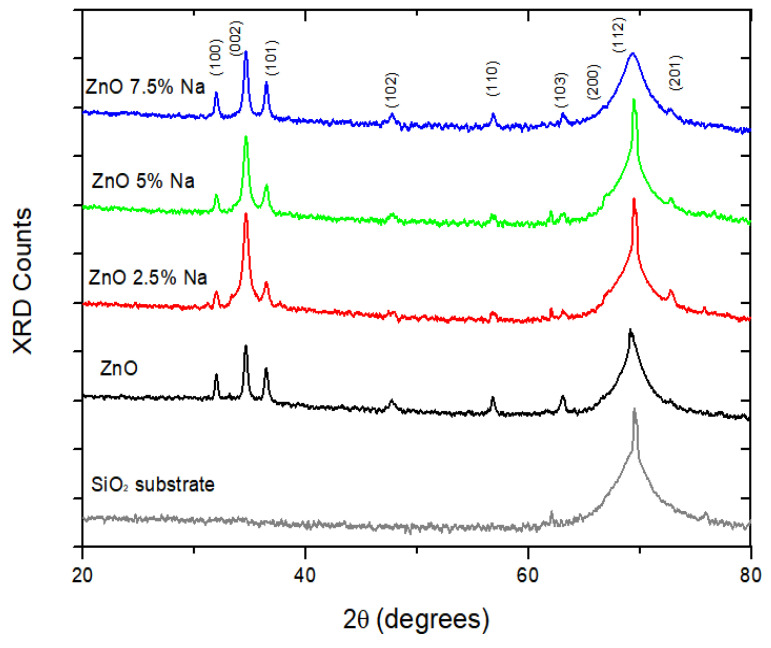
XRD patterns of the SiO_2_ substrate and the pure and Na-doped ZnO thin films.

The X-ray diffraction patterns of undoped and Na-doped ZnO thin films are compared in Figure 2.

The detected diffraction peaks, which were attributed to the (100), (002), (101), (102), (110), (103), (200), (112), and (201) facets, are consistent with the ZnO wurtzite hexagonal crystal structure (JCPDS 36-1451). The high and wide peak that arose at 2θ~69.56 was attributed to the Si substrate, probably due to the small thickness of the sensing films, which permitted the X-ray to penetrate the sensing layer and reach the substrate. All the samples showed the preferred orientations along the facets (100), (002), and (101). The doping with Na did not result in extra diffraction peaks except for those of pure ZnO, but clearly intensified the c-axis (002) and decreased the intensity of (100) and (101) facets, especially at lower Na concentrations (2.5%). This was also observed in ZnO thin films doped with calcium [21], aluminum, and magnesium [30]. The incorporation of Na led to a slight shift of the diffraction peaks to higher 2θ values (from 34.63 to 34.65) because of the larger ionic radius of Na (0.95 nm) compared to Zn (0.74 nm), thus forming lattice defects [21]. In addition, the average crystallite size (d) of ZnO thin films decreased from 39 nm to 35 nm and 34 nm with the incorporation of 2.5% and 5% Na, respectively. However, the further increase in dopant concentration (7.5% Na) increased the crystallite size to 38 nm. The non-linear change of crystallite size depending on the percentage of the dopant was also reported in a study by Altun et al. (2021) [12].

### 3.2. Field Emission-Scanning Electron Microscopy (FE-SEM)

The results of the structural analysis performed by FE-SEM are presented in Table 2. As observed, both ZnO and Na-doped ZnO thin films consisted of dense grains from agglomerated nanoparticles. Doping with Na did not significantly change the morphology or the mean particle size, which were statistically similar. Regarding the size distribution, 2.5% Na-doped films exhibited a wider range (24.4–114.7 nm), with most of the particles, however, being well concentrated in the 47–58.3 nm region, in contrast to the pure ZnO films, which had a slightly narrower spectrum but the majority of the grains were in a larger range (35.8–73.4 nm). This phenomenon was also observed in previous research by Benzitouni et al. (2017) and was attributed to the formation of grain aggregates due to the higher mobility caused by doping [48]. The growth process of large particles is known as Ostwald ripening: large particles grow at the expense of small particles [49]. The ripening process was even more evident in films with a higher doping percentage (7.5% Na), where several possibly aggregated grains with a size of 101.6–112.2 nm appeared. Finally, it was observed that after 2.5% Na doping, the film thickness increased by about 30 nm. As the doping percentage increased, the films became thicker, with the 7.5% Na-doped ZnO films showing the highest thickness.

### 3.3. Atomic Force Microscopy (AFM)

The two- and three-dimensional topography images, as well as the root mean square (RMS) roughness for the sensor films developed in this research, are shown in Table 3.

In general, all the sensing films showed the characteristic surface of ZnO, which was composed of spherical particles at various scales. Comparing the topographies of the plain ZnO film and the Na-doped films, the latter exhibited a smoother surface, which was confirmed by the reduction of the RMS surface roughness. The creation of a smoother structure and surface after doping was also highlighted in the research of Benzitouni (2017) and Banerjee (2010), where cobalt and aluminum were incorporated into the ZnO film, respectively [48,50]. In fact, in both studies, it was concluded that the roughness depended, to a significant extent, on the percentage of the dopant, and that while the doping with a small percentage led to a significant reduction of the RMS roughness, the incorporation of larger percentages led to an increase of its value, as was the case in the present study.

The combination of the results of the two microscopy methods, FE-SEM and AFM, led to the conclusion that the surface uniformity was strongly influenced by the thickness of the thin film and the arrangement of the grain particles. Increasing the film thickness and its particle size led to a better surface uniformity and a decrease in the roughness value [51]. Xu (2012) reported similar results on the relationship between ZnO thin film thickness and RMS surface roughness values [52].

### 3.4. CO_2_ Measurements

The sensing response of the developed sensors is shown in Table 4. The response of the sensors was evaluated for various CO_2_ values (50, 125, 250, 330 and 500 ppm) under a constant voltage of 2 V and at room temperature (25 °C). During the experimental procedure, a constant voltage was applied to the sensor terminals while the sensor current value and time were recorded. The measurement began at 0% CO_2_ concentrations within the chamber; then with an appropriate combination of flow through the two controllers, the CO_2_ concentrations were increased. After each CO_2_ step, a zero concentration step followed so that the sensor response and recovery time could be subsequently calculated. The net response (net signal) of the four developed sensors was determined by establishing a baseline at 0% CO_2_ concentration and subtracting it from the recorded signals at different CO_2_ levels. 

**Figure 3:**
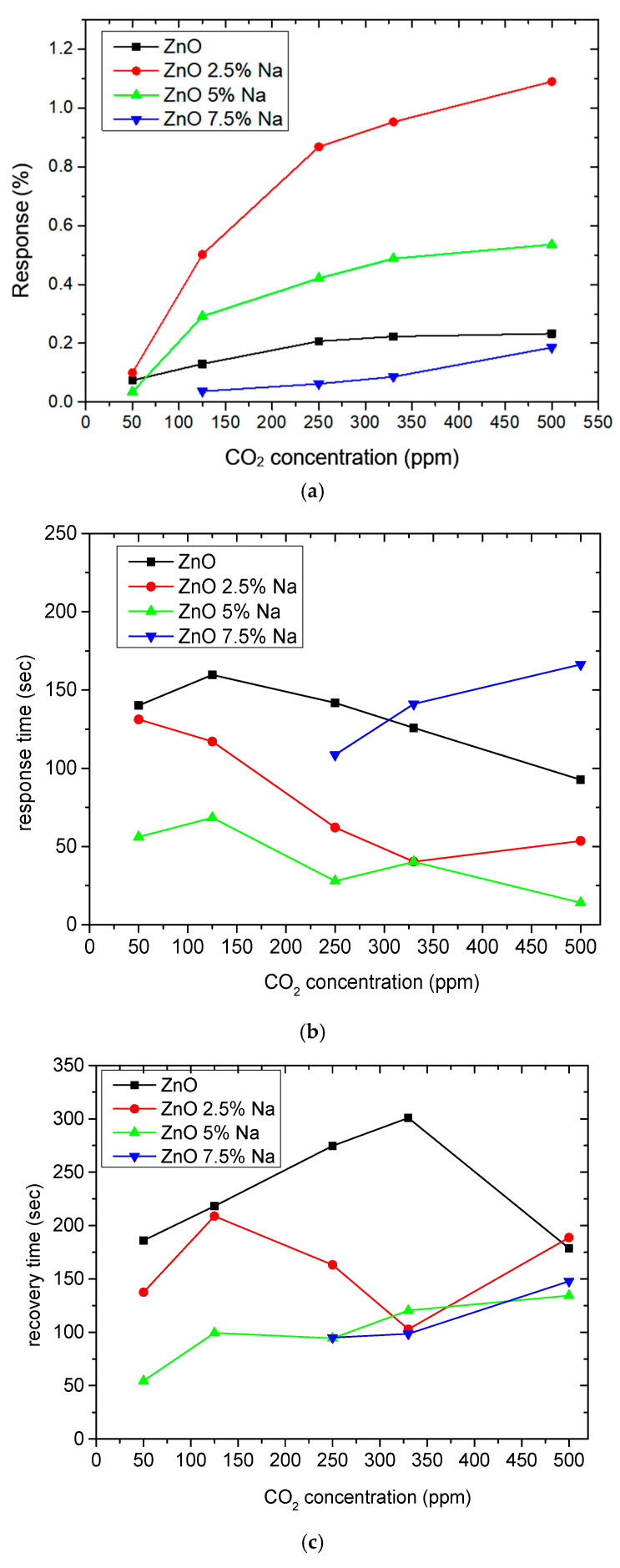
(**a**) Response, (**b**) response times, and (**c**) recovery times of pure ZnO, 2.5% Na-doped, 5% Na-doped, and 7.5% Na-doped sensing films.

The response of the sensors, as well as the response and recovery times as a function of CO_2_ concentration, are shown in Figure 3.

In general, the main mechanism of CO_2_ gas detection by ZnO is based on the adsorption of CO_2_ molecules on the ZnO surface, resulting in a change in the electrical resistance of the surface [53].

More specifically, in the presence of atmospheric air, oxygen molecules (O_2_) are adsorbed on the ZnO surface, transferring electrons (e^−^) from its conduction band and forming oxygen ions O_2_^−^, O^−^, and O^2−^, depending on the temperature (low, intermediate, or high). This results in the electron concentration decreasing and an electron depletion zone being created on the surface of each ZnO molecule [28]. The following equations describe the whole process as a function of temperature [54]:

O_2 (gas)_ → O_2 (adsorbed)_ (3)

O_2 (adsorbed)_ + e^−^_(surface)_ ↔ O_2_^−^_(adsorbed)_ (<100 °C) (4a)

O_2 (adsorbed)_ + 2e^−^_(surface)_ ↔ 2O^−^_(adsorbed)_ (100–300 °C) (4b)

O^−^_(adsorbed)_ + e^−^_(surface)_ ↔ O^2−^_(adsorbed)_ (>300 °C) (4c)

When the ZnO surface is exposed to CO_2_ gas, the adsorbed anions O_2_^−^, O^−^, and O^2−^ react with the CO_2_ molecules, which extract electrons from the conduction band of ZnO and form carbonate compounds, such as (CO_3_)^2−^ [55]. Consequently, chemical bonding and charge transfer occur between the metal oxide surface and the adsorbed gas. Since ZnO is an n-type semiconductor, charge transfer reduces the concentration of electrons on the ZnO surface, leading to an increase in the electrical resistance of the sensor, as shown in Table 4 [56]. The second phase of the process occurs according to the following Equations [57]:

CO_2 (gas)_ + O^−^_(adsorbed)_ → (CO_3_)^2−^_(adsorbed)_ (5)

(CO_3_)^2−^_(adsorbed)_ → CO_2 (gas)_ + ½ O_2(gas)_ (6)

As seen in Table 4, the response of the sensors increased by increasing the CO_2_ content. The exact dependence of the sensor response on the different CO_2_ levels can be found in the equation shown in each figure of the response/CO_2_ concentration. In general, it was noticed that as the CO_2_ concentration increased, the response reached a relative plateau, especially in the cases of the sensors with 2.5 and 5% Na.

Comparing the performance of the plain ZnO sensor and the sensors with 2.5% and 5% Na, it was observed that the doping with minor concentrations of dopant contributed to an obvious improvement in the sensitivity and in the response and recovery times. Especially the sensor with 2.5% reduces the signal noise at zero CO_2_ concentration, whereas the sensor with 5% Na shortens the response and recovery times the most, which is a favorable effect for sensors. These improvements are due to the fact that by adding Na^+^ ions the microstructure of the pure ZnO changes and the conductivity increases. ZnO has a narrow hexagonal lattice, which is “open” to some extent since Zn atoms occupy half of the tetrahedral sites and all the octahedral sites are empty. Therefore, ZnO offers many sites to “accommodate” intrinsic defects and extrinsic impurities. When Na^+^ ions replace zinc Zn^2+^ sites, oxygen vacancies (VO•) are created. CO_2_ is then adsorbed through the formation of Na_2_CO_3_. The above-mentioned changes are described by the following Kröger Vink Equations:

Na^+^ ion substitution for Zn^2+^ in the lattice:

(7a) Na→Na’ZN+V

CO_2_ adsorption and formation of Na_2_CO_3_:

(7b) $$Na+{CO}_{2}\left(absorbed\right)\to {{Na}_{2}CO}_{3}$$

CO_2_ adsorption and interaction with oxygen vacancies:

(7c) CO2+VO•↔CO•2+VOx

Ionization of oxygen vacancies:

(7d) $${VO}^{•}↔{VO}^{••}+e’$$

where, O: oxygen, V: vacancy, ^•^: positive charge and e′: negative charge.

Therefore, replacing Zn^2+^ with Na^+^ in the ZnO crystal lattice can create additional oxygen vacancies ${VO}^{•}$ and defects, with Na-ZnO containing heavier concentrations of oxygen vacancies than ZnO. Subsequently, these additional vacancies increase electron concentration and CO_2_ adsorption, which enhances the response of the doped sensor and enables satisfactory detection of CO_2_ at room temperature [23]. The enhancement of sensing performance by 2.5% Na-doped ZnO sensor can be also attributed to the smaller size of the crystallites estimated via the XRD analysis results, which was also observed in the work of Petrov et al. (2021) [58].

Furthermore, the doping with a higher percentage of Na causes difficulty in detecting low amounts of CO_2_ since the sensor with 7.5% Na was not able to detect the amounts of 50 and 125 ppm of CO_2_. This is due to several factors, which are confirmed by FE-SEM and AFM morphology. Doping with 5 and 7.5% Na leads to a minimal increase in particle size due to agglomerations and a significant increase in film thickness, thus reducing both the CO_2_ adsorption sites and the surface-to-volume ratio. Therefore, the CO_2_ gas adsorption on the ZnO film is impeded [34]. The deterioration of the sensor response when incorporating higher percentages of dopants in ZnO has been observed in various studies [12,31,32,59,60].

## 4. Conclusions

In conclusion, Na doping at a low concentration of 2.5% in ZnO significantly improved the CO_2_ sensing performance of ZnO films, making them effective for detecting microbial spoilage in food products at room temperature. While pure ZnO nanostructures can detect CO_2_ at high temperatures higher than 200 °C, the addition of Na overcame the limitation and had various effects. The 2.5% Na-doped ZnO sensor demonstrated the most favorable properties, including reduced crystallite size, increased film thickness, and a smoother, more uniform surface structure. Finally and most importantly, Na doping at 2.5% and 5% enhanced the sensor performance by allowing the detection of very low concentrations of CO_2_, even 50 ppm, increasing the sensitivity and reducing the response and recovery times, while eliminating the “noise”. Overall, the developed sensors showed satisfactory CO_2_ sensing properties, with the 2.5% and 5% Na doped sensors having the best performance. These results confirm that the developed sensors can successfully be integrated into a smart packaging system, providing a real-time and reliable method to monitor freshness in food products at room temperature.

## Figures and Tables

**Table 1:** ZnO thin films manufactured for CO_2_ sensing: fabrication process, dopant concentrations, and sensing properties: optimum response and response/recovery time at CO_2_ concentration and temperature (T).

Fabrication Process	Doping	CO_2_ Concentration	Topt (°C)	Sensing Response	Response/Recovery Time	Ref.
direct current reactive magnetron sputtering	-	8.5 mbar	100	2.17	5 s/10 min	[34]
successive chemical solution deposition	1% Al	1000 ppm	25	~22.5	20 s/150 s	[35]
chemical bath deposition		2600 ppm	300	~17%	-	[28]
direct current reactive magnetron sputtering	-	1000 ppm	300	1.13%	20 s/20 s	[36]
sol-gel	5% In	100 ppm	200	16%	-	[31]
sol-gel	5% Ca	2500 ppm	450	113	10 s/10 s	[37]
spray pyrolysis	-	1000 ppm	200	~650	1.	[38]
radio-frequency magnetron sputtering	-	15%	300	9.978	57 s/107 s	[39]
aerosol spray pyrolysis	0.1 at. % Mn	100 ppm	25 (under UV)	66%	1.	[32]
sol-gel	2.5 at. % Na	50 sccm	25	81.9%	282.73 s/472.3 s	[23]
sol-gel	3% Ca/1% Al	5% CO_2_	300	5	2.	[11]
sol-gel	5% Ca	2500 ppm	450	~70	3.	[40]
chemical spray pyrolysis	-	400 ppm	350	65%	75 s/108 s	[33]
chemical bath deposition	-	15%	300	9	~75 s/~150 s	[41]
spray pyrolysis	-	100 Torr	150	~37	-	[42]
wet chemical synthesis	5% Ca	50,000 ppm	350	53%	-	[43]
wet chemical synthesis	5% Ca	25,000 ppm	350	~32%	-	[21]
sol-gel	5% Al/1% Mg	2000 ppm	200	1.9	-	[30]
chemical bath deposition	3% Cd	100 ppm	125	88.24%	11 s/10 s	[12]
wet chemical solution deposition	ZnO/CuO	2500 ppm	375	47%		[44]
in-situ annealing	2.4 at. % N	500 ppm	-	5	-	[45]
sol-gel spin coating	4.0 at. % La	200 SCCM	25	122.71%	24.4 s/44 s	[19]
sol-gel spin coating	5% Al	100 ppm	227	100%	90 s/160 s	[20]
sol-gel spin coating	-	200 ppm	450	~150	20 s/40 s	[46]

**Table 2:** Morphology, film thickness, particle size distribution, mean particle size and mean film thickness of: (1) pure ZnO, (2) 2.5% Na-doped, (3) 5% Na-doped and (4) 7.5% Na-doped thin films. FE-SEM top view and cross section photos are presented in 2.5 kV voltage, x100,000 magnification, 3 mm working distance (WD), and 100 nm scale bar.

	Top View	Cross Section	Size Distribution	Mean Particle Size (nm)	Mean Film Thickness (nm)
1	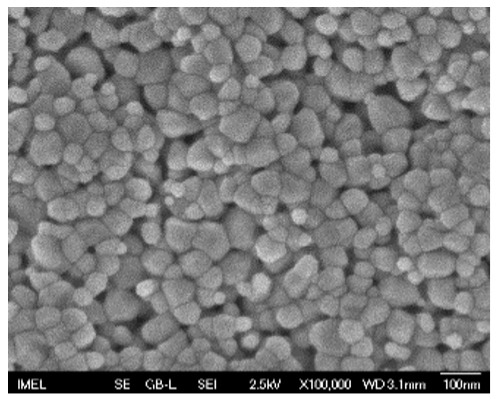	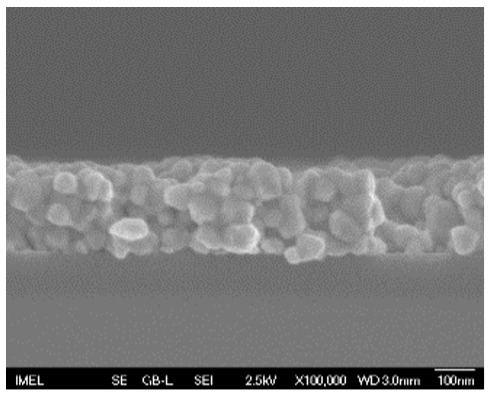	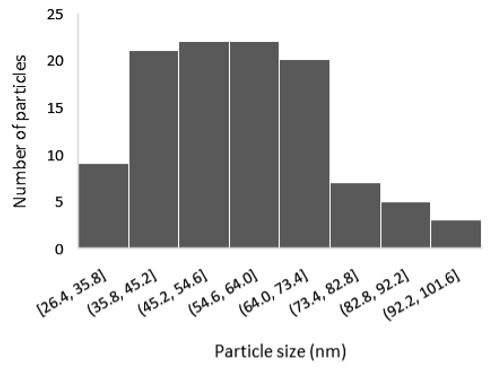	56 ^a^ ± 16	217 ^a^ ± 8
2	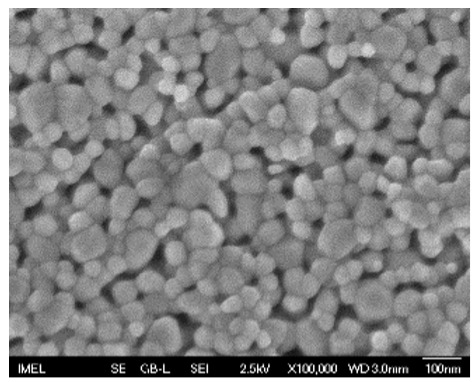	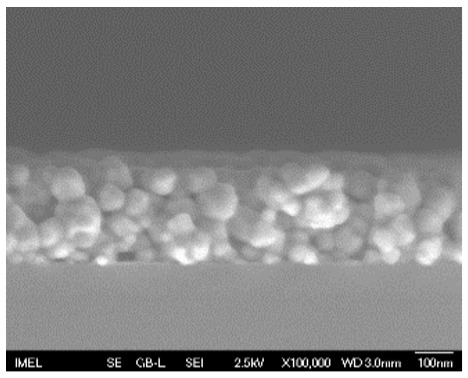	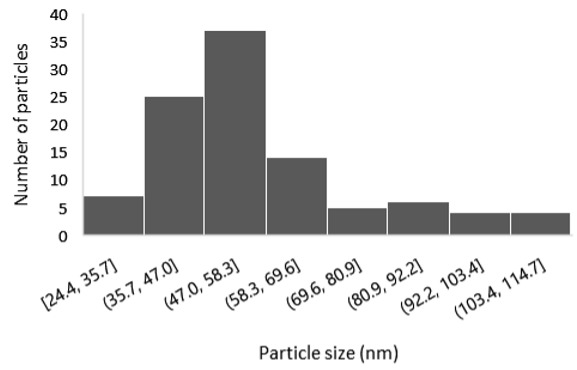	57 ^a^ ± 19	247 ^b^ ± 10
3	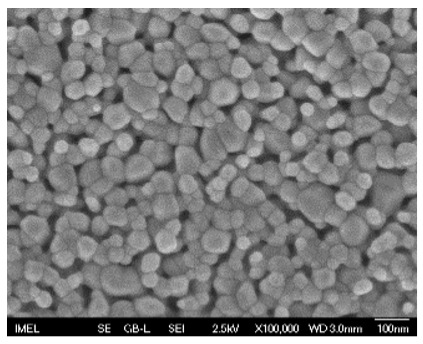	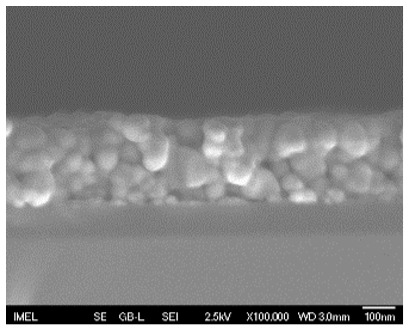	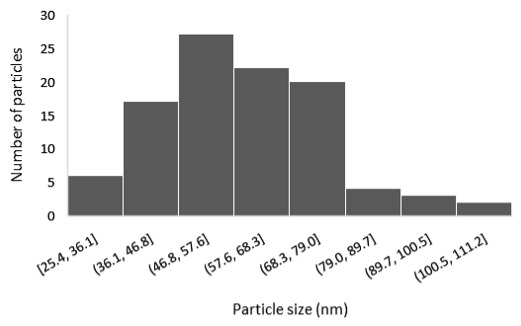	59 ^a^ ± 16	268 ^c^ ± 10
4	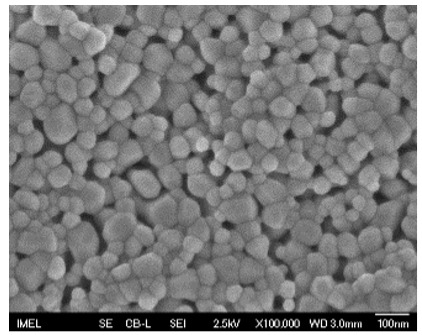	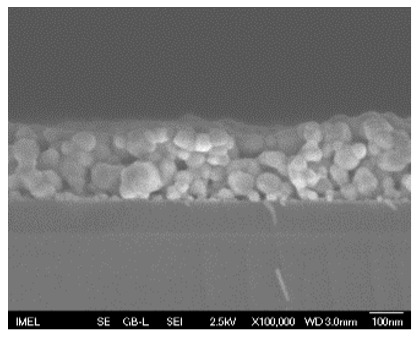	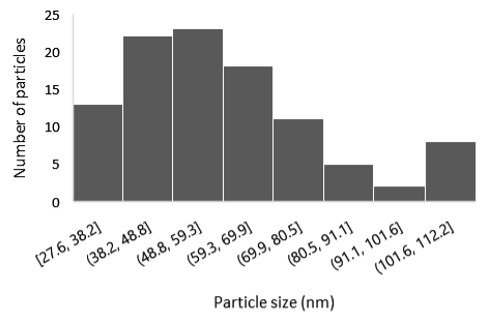	60 ^a^ ± 20	270 ^c^ ± 13

+/− shows the standard deviation between the replicates. Values not sharing the same superscript are significantly different (*p* < 0.05).

**Table 3:** AFM 2D and 3D topographies and RMS roughness of: (1) pure ZnO, (2) 2.5% Na-doped, (3) 5% Na-doped, and (4) 7.5% Na-doped thin films.

	2D Topography	3D Topography	RMS Roughness sq (nm)
1	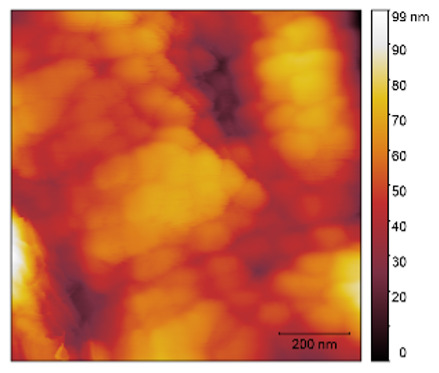	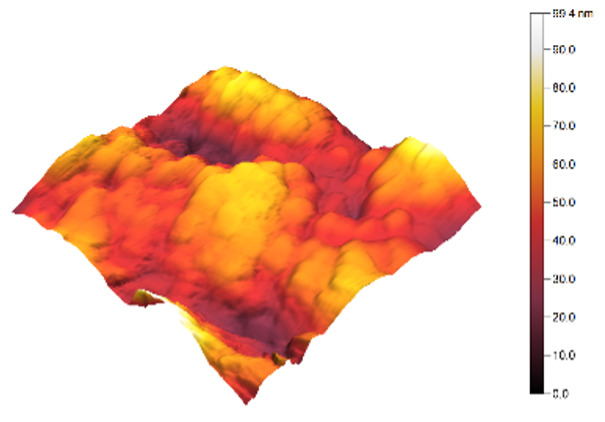	12.3
2	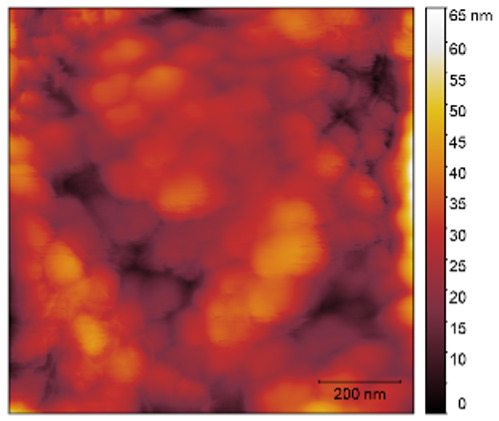	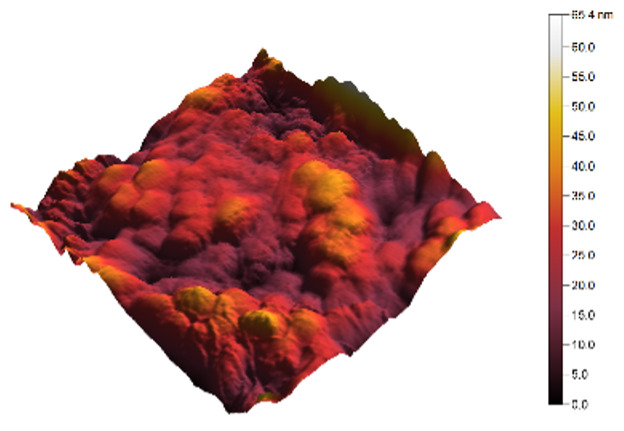	7.7
3	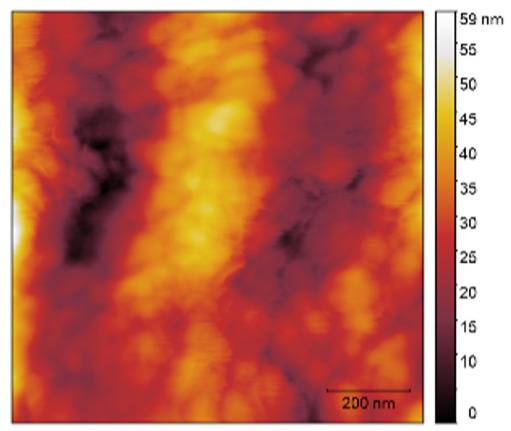	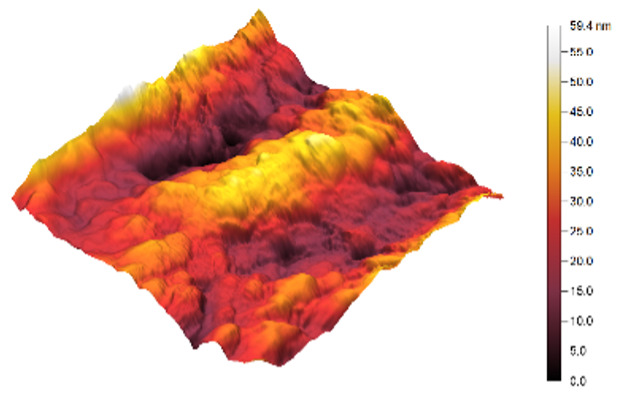	9.0
4	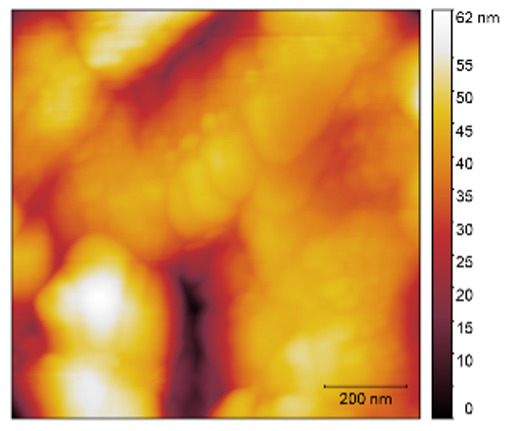	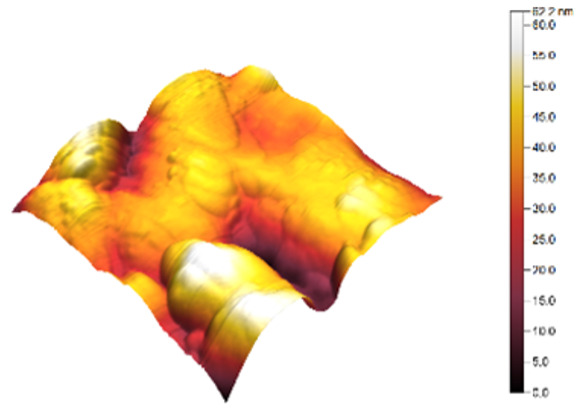	9.2

**Table 4:** Sensing response represented as Net Signal current (A) over time and at varying CO_2_ concentrations with (1) pure ZnO, (2) 2.5% Na-doped, (3) 5% Na-doped, and (4) 7.5% Na-doped thin films.

	Sensor Response (A)—Time (min)	Sensor Response (A)—CO_2_ Concentration (ppm)
1	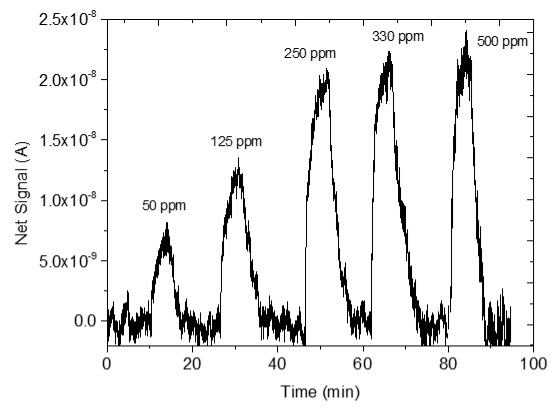	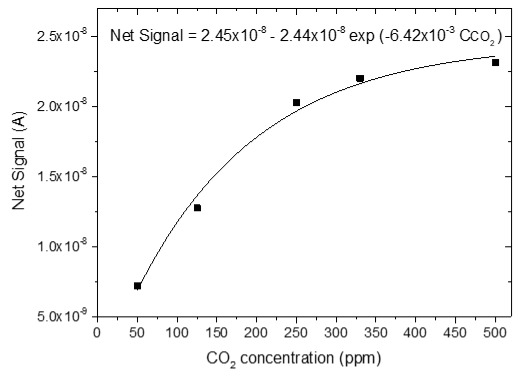
2	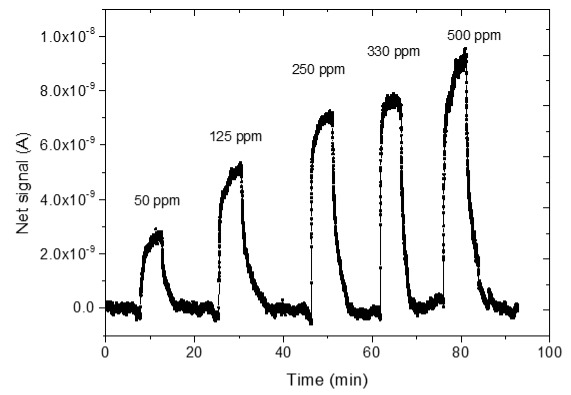	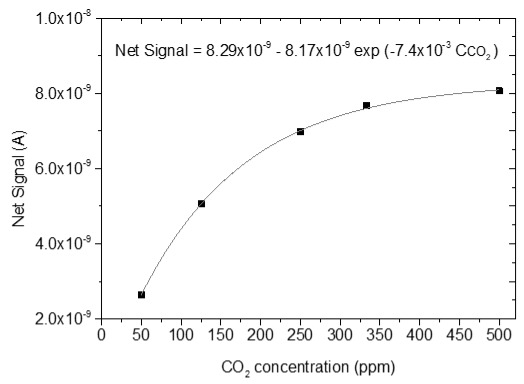
3	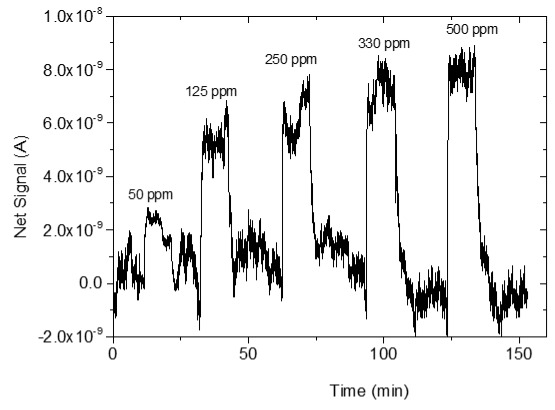	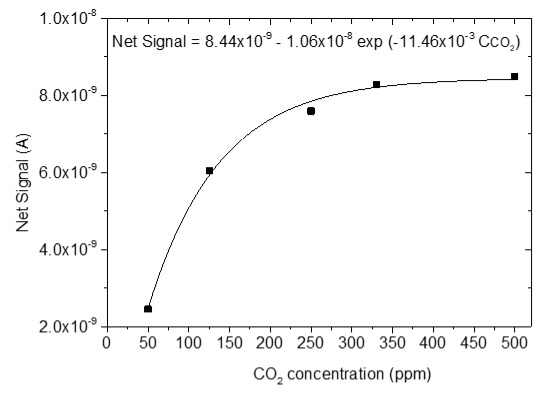
4	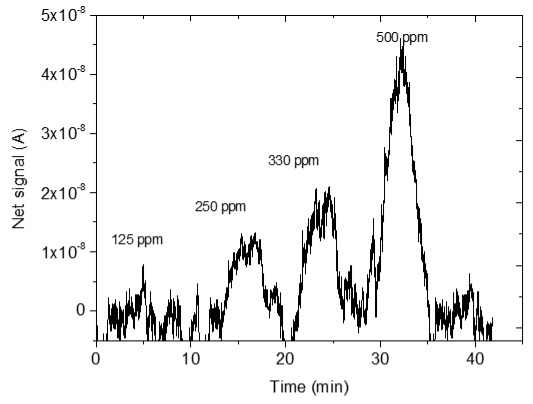	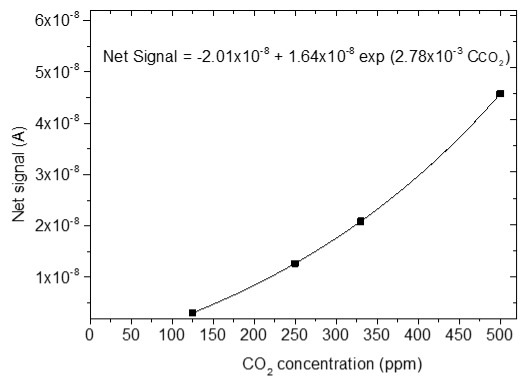

## Data Availability

The data presented in this study are available on request from the corresponding author (Marina Stramarkou’ mstrmarkou@chemeng.ntua.gr).
